# Supplementary material for: Identification of Freezing-Responsive microRNAs and Their Targets in Chinese Jujube by Small RNA and Degradome Sequencing
Source: Int J Mol Sci. 2026 Sep 4;27(17):7912. doi: 10.3390/ijms27177912 (PMC13566890; doi:10.3390/ijms27177912)
Supplement: Supplementary file 1 [file ijms-27-07912-s001.zip › Supplementary Material S2/novel_miR_210.pdf]

Provisional ID	:	novel_miR_210
Score total	:	0.3
Score for star read(s)	:	-1.3
Score for read counts	:	0
Score for mfe	:	0
Score for randfold	:	1.6
Score for cons. seed	:	
Total read count	:	64
Mature read count	:	60
Loop read count	:	4
Star read count	:	0

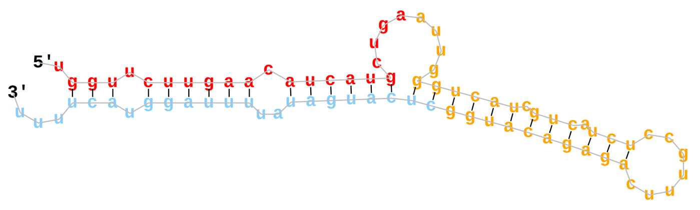

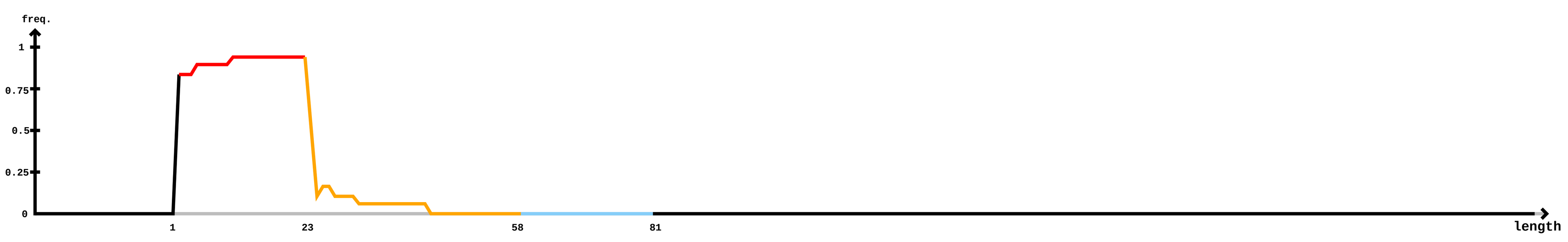

Mature Star

```
5'- cauucauacaauuugaucucucugguucuugaacaucaugcugaauugggucaucgucaucuccguuucagagacauggcucaugauauuuuagguacuuuuuucuuuuuuauuuccuucaauuaauuaucuauucguuuuccuuuucauucaacuuuuuagggaaauuucauuugguaacccucuuucaagggugaaauuggaaaacgaucucaauuuucaaaauuuuauaauuuuaaccccuauuuua  -3'   exp
    ...........(((((.......(((.((((((.((((((........((((((.(((.(((.......))))))))))))))))))..)))))).))).................................((((((((.((((((((........((((..(((......)))..))))......))))))))..)))))))))........)))))..........................  reads  mm  sample
    ......................ugguucuugaacaucaugcuga.............................................................................................................................................................................................................  33  0  LCK-1
    ......................ugguucuugaacaucaugcuga.............................................................................................................................................................................................................  23  0  LCK-2
    .........................uucuugaacaucaugcugaauug.........................................................................................................................................................................................................  4  0  JCK-1
    ...............................aacaucaugcugaauuggguc.....................................................................................................................................................................................................  3  0  LT-2
    ..............................................ugggucaucgucaucucc.........................................................................................................................................................................................  4  0  LT-2
```
